# Gut microbiota and chronic obstructive pulmonary disease: a Mendelian randomization study

**DOI:** 10.3389/fmicb.2023.1196751

**Published:** 2023-06-19

**Authors:** Yi Wei, Xuechao Lu, Chao Liu

**Affiliations:** ^1^Department of Chinese Medicine, Shandong University of Traditional Chinese Medicine, Jinan, China; ^2^Department of Respiratory and Critical Care Medicine, Qingdao Traditional Chinese Medicine Hospital (Qingdao Hiser Hospital), Qingdao, China; ^3^Department of Medical Imaging, Qingdao Traditional Chinese Medicine Hospital (Qingdao Hiser Hospital), Qingdao, China

**Keywords:** gut microbiota, chronic obstructive pulmonary disease, Mendelian randomization, causal association, genome-wide association study

## Abstract

**Background:**

A growing number of studies implies a strong association between gut microbiota and chronic obstructive pulmonary disease (COPD). However, the causal impact between gut microbiota and COPD remains unclear. As a result, we used a two-sample Mendelian randomization (MR) method to investigate the connection between gut microbiota and COPD in this study.

**Methods:**

The largest available genome-wide association study (GWAS) of gut microbiota was obtained from the MiBioGen consortium. Summary-level dataset for COPD were obtained from the FinnGen consortium. The main analysis method for determining the causal link between gut microbiota and COPD was inverse variance weighted (IVW). Subsequently, pleiotropy and heterogeneity tests were performed to determine the reliability of the results.

**Results:**

IVW method identified 9 bacterial taxa nominally associated with the risk of COPD. Class Actinobacteria (*p* = 0.020), genus *Allisonella* (*p* = 0.024), genus *Coprococcus2* (*p* = 0.002) and genus *Oscillospira* (*p* = 0.018) were protective against COPD. In addition, order Desulfovibrionales (*p* = 0.011), family Desulfovibrionaceae (*p* = 0.039), family Peptococcaceae (*p* = 0.020), family Victivallaceae (*p* = 0.012) and genus *Marvinbryantia* (*p* = 0.017) were associated with a higher risk of COPD. No pleiotropy or heterogeneity were found.

**Conclusion:**

According to the findings of this MR analysis, a causal relationship exists between certain gut microbiota and COPD. New insights into the mechanisms of COPD mediated by gut microbiota are provided.

## 1. Introduction

Chronic obstructive pulmonary disease (COPD) is a progressive inflammatory disease of the lungs with pathological changes in both large and small airways (Divo et al., 2014; May and Li, 2015). COPD has become the third leading cause of death worldwide, with an increasing incidence with age (Raherison and Girodet, 2009). COPD is reported to be 2–3 times more common in older adults (Chapman et al., 2006; Easter et al., 2020). The prevalence of COPD disease increases with age, and smoking is also an important risk factor for COPD (Ito and Mercado, 2014; Hikichi et al., 2019).

Not only is the gut microbiota engaged in the digestion of food and the absorption of nutrients, but it is also involved in the physiological regulation of the host through the creation of hormonally active chemicals (Kim and Jazwinski, 2018; Chen et al., 2021). Gut microbiota is a big and complicated population of microorganisms. It has been demonstrated that changes in the gut microbiota can have an effect on a variety of distant organs, including the lungs. The interaction that occurs between bacteria in the gut and germs in the lungs is referred to as the “gut-lung axis” (Budden et al., 2017). This axis, which connects the digestive tract and the pulmonary capillaries, makes it easier for endotoxins, microbial metabolites, cytokines, and hormones to enter the bloodstream (Dang and Marsland, 2019). The gut microbiota is thought to be extensively altered in COPD patients and to play a key role in the pathophysiology of COPD, according to expanding amounts of evidence (Krumina et al., 2022; Lai et al., 2022). Bowerman et al. (2020) recruited 28 COPD patients and 29 healthy controls and compared the two gut microbiotas and found that 146 bacterial species differed. At the family level, Eubacteriaceae, Bifidobacteriaceae, Streptococcaceae and Veillonellaceae were enriched in the COPD group. Li et al. (2021) reported that, compared to the healthy population, the relative abundance of the phylum Bacillus mimicus in the gut microbiota of COPD patients was lower, while the relative abundance of the thick-walled phylum was higher, and Prevotella was enriched in COPD patients. The investigators also found lower levels of short-chain fatty acids (SCFA) in the COPD group. However, in observational studies, the relationship between gut microbiota and COPD is susceptible to confounding factors (such as age, smoking), and reverse causation. It is uncertain whether these associations are causal related.

Mendelian randomization (MR) is an epidemiology method uses integrated genome-wide association studies (GWAS) summary-level data to select eligible single nucleotide polymorphisms (SNPs) as instrumental variables (IV) for exploring the causal relationship between exposure and outcome (Bowden and Holmes, 2019; Birney, 2022). The advantage of this approach is that genetic variants are randomly assigned at the time of conception and therefore are not subject to the same biases and confounders as in traditional observational studies (Davey Smith and Hemani, 2014). The MR approach has seen widespread application in the investigation of potential causal connection between gut microbiota and a variety of disorders affecting several body systems, such as cardiovascular diseases (Jia et al., 2019; Luo et al., 2022), metabolic diseases (Liu et al., 2022), neurological diseases (Zhuang et al., 2020), and autoimmune diseases (Xiang et al., 2021; Xu et al., 2021). The investigation of the causal connection between the gut microbiota and COPD not only increases our comprehension of the gut bacterial pathogenesis, but also makes it easier to develop personalized treatments for COPD by providing a more in-depth understanding of the interventions that can be made to the gut microbiota. It is of the paramount importance to get a deeper comprehension of the causal relationship that exists between COPD and the microbiota discovered in the gut. As a consequence, in this work, we carried out a two-sample MR analysis in order to investigate the potential causal connection between the microbiota in the gut and COPD. This is the first study that we are aware of that investigates whether or not there is a causal link between the gut microbiota and the development of COPD.

## 2. Materials and methods

### 2.1. Study design

**Figure 1:**
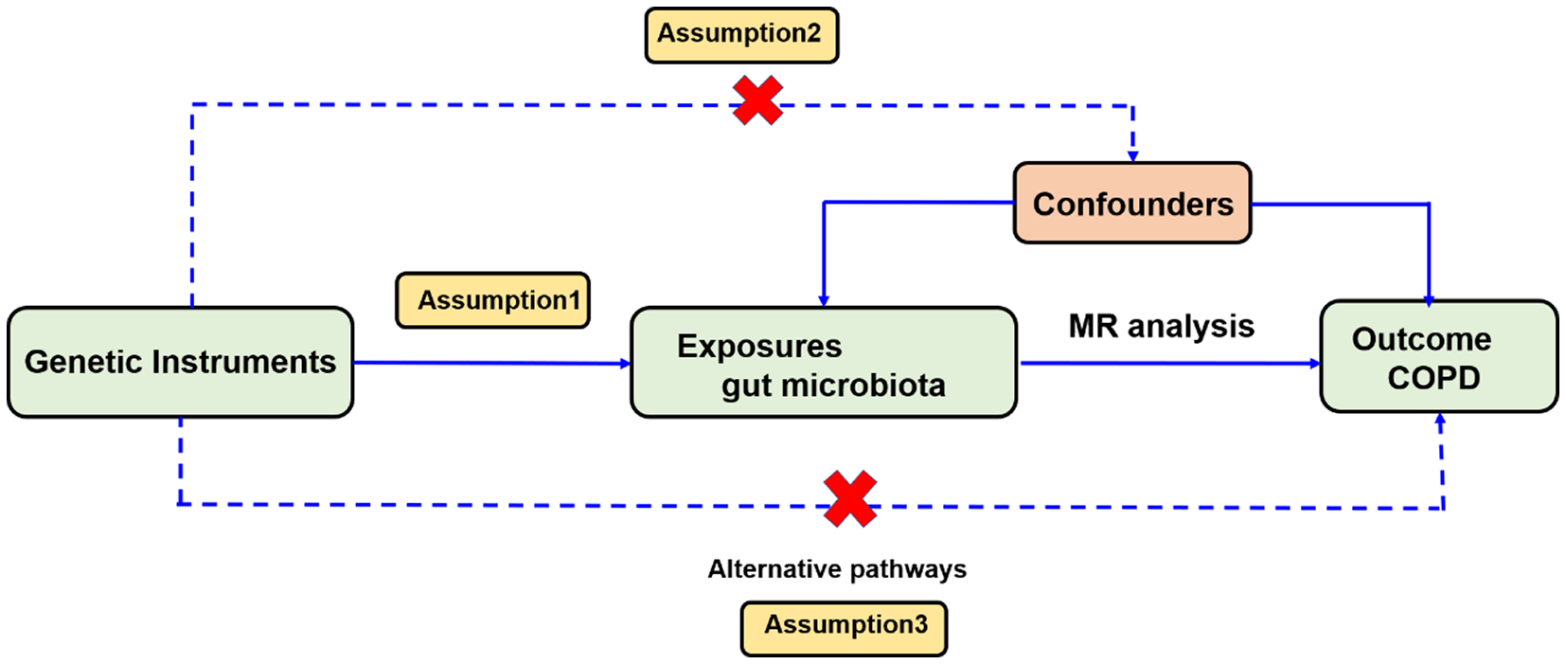
Overview of the Mendelian randomization analysis and three main assumptions.

We investigated the association between gut microbiota and COPD using a two-sample MR analysis method. To reduce the influence of confounding factors on the results, the MR approach should satisfy three key assumptions. (1) SNPs significantly associated with gut microbiota are selected as instrumental variables (IVs); (2) IVs are independent, which means they are not associated with other confounding factors (e.g., age, smoking); and (3) IVs are only associated with the outcome through exposure and should not influence the outcome through other pathways (Figure 1).

### 2.2. Ethics statement

Summary-level data for the studies used for analysis were composed and obtained from published studies. All original studies were conducted in accordance with the Declaration of Helsinki and were conducted with the approval of the relevant ethics committees (MiBioGen Consortium and FinnGen research consortium). This study only used publicly available summary-level data from published studies, therefore did not require additional ethical approval. In addition, this study was performed in strict compliance with STROBE-MR guidelines.

### 2.3. Data source

Summary-level data for the human gut microbiota were obtained from the largest GWAS published to date. The meta-analysis was conducted by the MiBioGen consortium^1^ which included 18,340 subjects from 24 cohorts in 11 countries. A total of 211 bacterial taxa units were included, involving 131 genera, 35 families, 20 orders, 16 classes and 9 phyla (Kurilshikov et al., 2021).

Summary statistics for COPD were retrieved from a dataset in that were deposited in the FinnGen biobank analysis round 5, comprising 6,915 COPD cases and 186,723 controls.

### 2.4. Selection of IVs

First, based on previous thresholds for screening SNPs, we selected SNPs with a significance threshold of *p* < 1.0 × 10–5 to be selected as potential IVs to obtain more comprehensive results. Afterward, with reference to previous studies, we clumped SNPs to achieve independent loci, setting the threshold of the linkage disequilibrium (LD) at *r*^2^ = 0.1 and clumping window = 500 kb. Subsequently, it was necessary to ensure that the effect of SNPs on exposure corresponded to the same alleles as the effect on outcome, so as a matter of principle, echo SNPs were not counted as IVs. In terms of the relationship between gut microbiota and COPD, COPD-related characteristics or risk factors, such as smoking, age, are most likely to be potential and substantial confounders. In order to verify the second MR assumption, we consulted the PhenoScannerV2 database and searched about each IV as well as its proxy features. Then we removed SNPs related to confounding factors. Next, we extracted the SNPs that had been screened for surrogating gut microbiota indicators from the COPD summary-level GWAS dataset. In case of one or more SNPs absent in the COPD GWAS database, we would not use any proxy instruments for these missing SNPs.

To check whether estimates of the effect of causality might be affected by weak instrument bias, the strength of IVs was tested using the *F* statistic. No significant weak instrumental bias is considered to exist if the corresponding *F*-statistic >10.

### 2.5. MR analysis

The relationship between gut microbiota and COPD was first assessed using inverse variance weighted (IVW) as the primary MR method, which incorporates the Wald estimator of SNPs to estimate the effect. The results of the IVW method will be plausible if each SNP satisfies the assumption of MR (level-free pleiotropy). In addition, MR-Egger, weighted median, simple mode and weighted mode were used as supplementary analysis methods. Using the Bonferroni correction, we established significance thresholds for MR results at each of the five taxonomic levels. The Bonferroni correction threshold for each feature level is 0.05/n (where n is the number of independent bacterial taxa at the corresponding taxonomic level). MR results can be considered significant when the *p*-value is less than the Bonferroni correction threshold. Also, we regarded *p* < 0.05 as nominally significant.

To assess the robustness of the results, further multiple sensitivity analyses were conducted. The Cochran’s Q statistic of the IVW method and the MR-Egger regression method were used to quantify the heterogeneity of IVs (considering *p* < 0.05 as possible heterogeneity of IVs). The leave-one-out sensitivity analysis is used to test the stability of outliers and results. The MR-Egger and MR Pleiotropy Residual Sum and Outlier (MR-PRESSO) tests are used to test for pleiotropy and outliers. MR-Egger intercept approach is applied for the purpose of particularly determining whether or not horizontal pleiotropy is present. In the absence of considerable horizontal pleiotropy, a *p*-value greater than 0.05 shows this fact. In comparison to MR-Egger, MR-PRESSO possesses a greater level of accuracy and assists in the detection of horizontal pleiotropy as well as outliers.

## 3. Results

### 3.1. Characteristics of SNPs

We screened 211 bacterial taxa IVs separately. a total of 2,788 IVs reached a locus-wide significance level (*p* < 1 × 10–5). A total of 2,561 SNPs were associated with COPD after excluding the effect of LD in specific bacterial groups. Details of the selected IVs are shown in Supplementary Table S1. The F-statistics of the IVs were all greater than 10, indicating that the estimates are unlikely to be affected by weak instrumental bias.

### 3.2. Causal effect of gut microbiota on COPD

**Figure 2:**
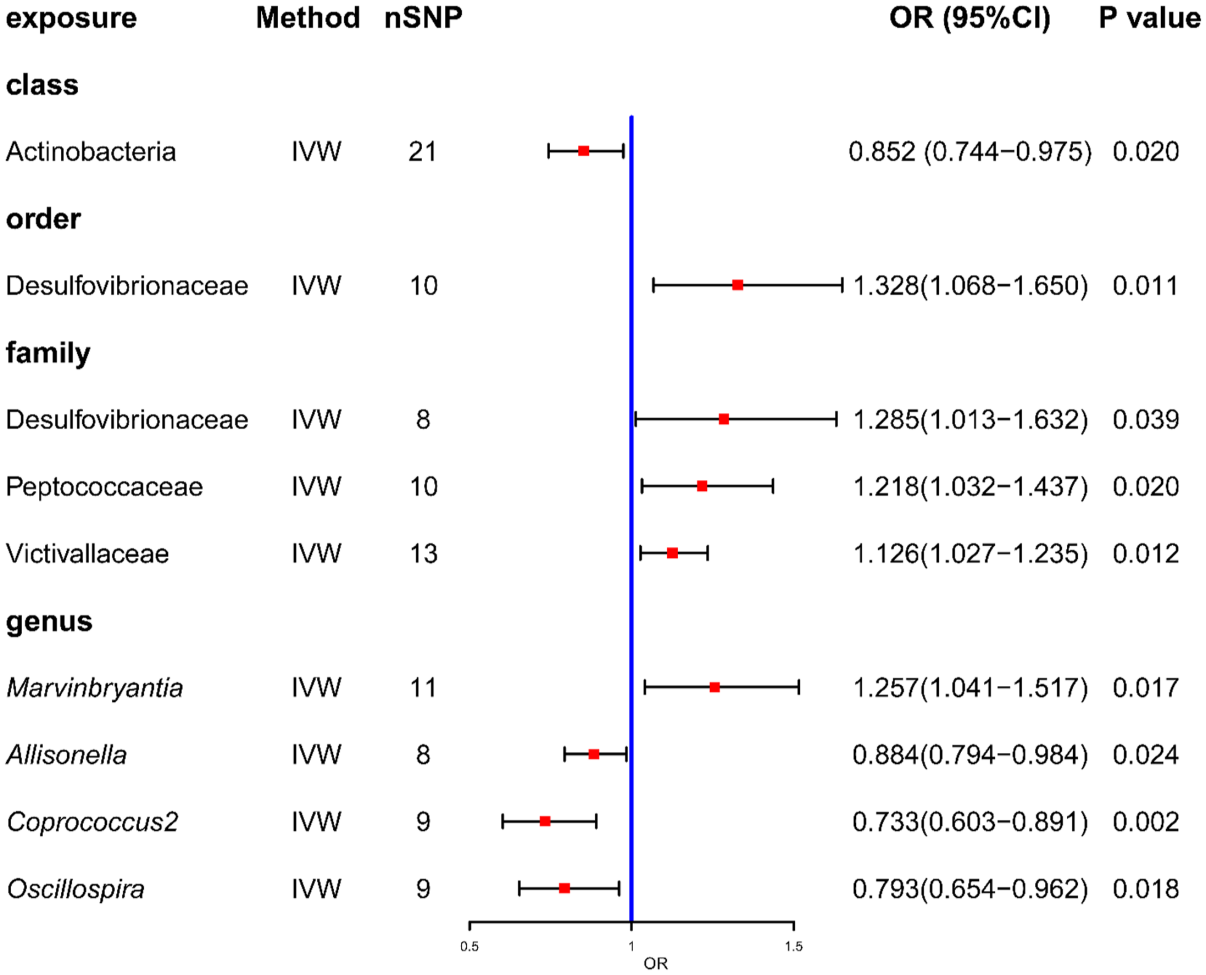
Forrest plot for summary causal effects of gut microbiota on COPD risk based on IVW method for the primary analysis. The forest plot demonstrates that class Actinobacteria, order Desulfovibrionales, family Desulfovibrionaceae, family Peptococcaceae, family Victivallaceae and genus *Marvinbryantia*, genus *Allisonella*, genus *Coprococcus2*, and genus *Oscillospira* have causal effect on COPD risk.

After MR analysis, the primary analysis IVW method showed that the relative abundance of the nine genetically predicted bacterial taxa was causally associated with COPD (Figure 2). And all the result were showed in Supplementary Table S2.

Specifically, at the Class level, Actinobacteria (odds ratio [OR] = 0.852, confidence interval [CI] = 0.744–0.975, *p* = 0.020) had a protective effect against COPD. At the genus level, a total of three gut microbiota were found to have a negative causal effect on the development of COPD. These included *Allisonella* (OR = 0.884, CI = 0.794–0.984, *p* = 0.024), *Coprococcus2* (OR = 0.733, CI = 0.603–0.891, *p* = 0.002) and *Oscillospira* (OR = 0.793, CI = 0.654–0.962, *p* = 0.018). In addition, at the order level, Desulfovibrionales (OR = 1.328, CI = 1.068–1.650, *p* = 0.011) may increase the risk of COPD. At the family level, Desulfovibrionaceae (OR = 1.285, CI = 1.013–1.632, *p* = 0.039), Peptococcaceae (OR = 1.218, CI = 1.032–1.437, *p* = 0.020) and Victivallaceae (OR = 1.126, CI = 1.027–1.235, *p* = 0.012) may be associated with a higher risk of COPD. At the genus level, higher abundance of *Marvinbryantia* (OR = 1.257, CI = 1.041–1.517, *p* = 0.017) similarly indicated a significantly higher risk of COPD. The above results are shown by scatter plots (Supplementary Figure S1) and forest plots for causal effects of gut microbiota on COPD risk with individual SNPs (Supplementary Figure S2). None of these MR results passed Bonferroni correction for multiple testing, but there were still *p* values <0.05, which can be considered as nominally significant.

The findings of the Cochrane’s Q test, which are presented in Table 1, indicated that there was no significant heterogeneity identified among the SNPs that were chosen (*p* > 0.05). According to the findings of the MR Egger test for pleiotropy, the results of our MR study did not demonstrate pleiotropy (*p* > 0.05; Table 2). The leave-one-out method suggested that some single SNPs might lead to some bias in genetic prediction (Supplementary Figure S3). However further MR-PRESSO analysis did not reveal any significant outliers (all *p* > 0.05 for global test). In addition, MR-PRESSO also showed that our MR analysis results did not show horizontal pleiotropy (all *p* > 0.05).

**Table 1:** The heterogeneity results from the Cochran’s *Q* test.

No	Level	Bacterial taxa	MR-Egger		IVW	
			*Q*	*p*-value	*Q*	*p*-value
1	Class	Actinobacteria	25.939	0.132	26.014	0.165
2	Order	Desulfovibrionales	4.692	0.790	4.986	0.836
3	Family	Desulfovibrionaceae	3.514	0.742	4.358	0.738
4	Family	Peptococcaceae	2.712	0.951	2.713	0.975
5	Family	Victivallaceae	9.129	0.610	9.258	0.681
6	Genus	*Marvinbryantia*	9.964	0.353	10.076	0.434
7	Genus	*Allisonella*	3.322	0.768	5.607	0.586
8	Genus	*Coprococcus2*	7.708	0.359	7.915	0.442
9	Genus	*Oscillospira*	8.425	0.297	9.328	0.315

**Table 2:** Pleiotropy results from Egger intercept analysis.

No	Level	Bacterial taxa	Egger intercept	SE	*p*-value
1	Class	Actinobacteria	−0.003	0.013	0.817
2	Order	Desulfovibrionales	−0.019	0.034	0.603
3	Family	Desulfovibrionaceae	−0.035	0.038	0.394
4	Family	Peptococcaceae	0.001	0.022	0.973
5	Family	Victivallaceae	−0.012	0.033	0.726
6	Genus	*Marvinbryantia*	−0.010	0.031	0.758
7	Genus	*Allisonella*	−0.079	0.053	0.181
8	Genus	*Coprococcus2*	−0.018	0.043	0.678
9	Genus	*Oscillospira*	−0.036	0.041	0.415

## 4. Discussion

This is the first large-scale comprehensive MR study to investigate the causal relationship between gut microbiota and COPD at the genetic prediction level. Previously, the relationship between gut microbiota and COPD has been investigated mainly through clinical trials and animal models (Wang et al., 2021). Several clinical studies have collected feces from COPD patients and analyzed the changes in patient flora by 16S rRNA gene sequencing (Ramsheh et al., 2021). However, these studies are susceptible to confounding factors (e.g., age, smoking), making it difficult to determine whether there is a causal association between gut microbiota and COPD. Therefore, the present MR study demonstrated that certain gut microbiota has a causal association with COPD risk. This may facilitate the discovery of new biomarkers in future COPD studies.

The microbiota that lives in human’s digestive system is a vital component of human life and plays an important part in the organization and performance of the human body (Zhang et al., 2020). The microbiota in the gut is responsible for the digestion of nutrients, the growth of the immune system, and the stimulation of a wide variety of host functions (Tan et al., 2020). Previous research has shown a significant amount of interest in the potential molecular pathways of the gut microbiota in the pathophysiology of COPD (Rajilić-Stojanović et al., 2007). It has been discovered that the microbial makeup of the gut plays a role in the development of COPD by playing a regulatory role in inflammation. COPD onset and disease progression are closely connected with inflammation (Qu et al., 2022).

In our study, a total of nine gut microbiota were found to have a nominal causal relationship with COPD. Four groups of bacteria are protective against COPD, including class Actinobacteria, genus *Allisonella*, genus *Coprococcus2*, genus *Oscillospira*. Actinobacteria are a diverse group of Gram-positive bacteria (Kim et al., 2021). Although Actinobacteria represent only a small fraction, they play a key role in maintaining intestinal homeostasis (Binda et al., 2018). Almost all Actinobacteria are involved in microbial homeostasis, some as probiotics and others as pathogens that cause inflammation. For example, *Bifidobacteriaceae*, which are classified as class Actinobacteria, are representative of the beneficial flora that have been shown to have a positive impact on intestinal health and immunity by helping to regulate the gut microbiome, promoting the growth of beneficial bacteria and reducing the number of harmful bacteria (Mattarelli et al., 2014). In addition, some genera of actinomycetes have been used as a source of natural antibiotics, such as streptomycin. Both *Coprococcus2* and *Oscillospira* are producers of short-chain fatty acids (SCFA)(Nagpal et al., 2018; Singh et al., 2022). SCFA are produced primarily through glycolytic fermentation of carbohydrates and are essential for maintaining metabolic and immune homeostasis. SCFA, and in particular its product butyrate, in particular, is an important substrate for maintaining intestinal integrity and has been shown to enhance intestinal barrier function by increasing the expression of the tight junction proteins claudin-1 and Zonula Occludens-1 (ZO-1)(Wang et al., 2012). In addition, butyrate has been found to limit the expression of the inflammatory cytokine interferon-gamma (IFN-γ) to improve the inflammatory response (Klampfer et al., 2003; Sun et al., 2017).

In total, five positive causal relationships were identified in this study, including order Desulfovibrionales, family Desulfovibrionaceae, family Peptococcaceae, family Victivallaceae and genus *Marvinbryantia* with COPD. Previous observational studies have found that bacterial abundance from family Peptococcaceae is significantly increased based on 16S rRNA gene sequencing of the fecal microbiome of COPD patients. This remains consistent with our findings. Desulfovibrionales and Desulfovibrionaceae contain sulfate-reducing genes, which reduce sulfate to H_2_S, disrupt the intestinal barrier, and produce endotoxins and pro-inflammatory cytokines, such as interleukin (IL)-6 (Weglarz et al., 2003). These cytokines can enter the bloodstream and affect distant organs, including the lungs. These molecules can further contribute to systemic inflammation and decreased lung function in COPD patients.

This study has several strengths. To begin, this is the first MR analysis using two samples to investigate the possible causal connection between gut microbiota and COPD. Traditional observational studies have a greater potential for bias due to the presence of confounding variables and the possibility of reverse causality. Second, the summary-level data on gut microbiota is the largest genome-wide association study (GWAS) to date, and the dataset is based on multiple human populations. This allows our findings to be generalized to a variety of human groups. In addition, the epidemiological impact of MR analysis is enormous and its use is likely to continue to grow in the coming years. As more genetic data become available and new methods are developed, MR analysis will continue to be a valuable tool for understanding the causal relationship between risk factors and disease outcomes.

Despite this, there are still certain restrictions on what can be concluded from this study. First, the use of summary statistics rather than raw data in the research made it impossible to do additional subgroup analyses, such as distinguishing between the various stages of COPD. Additionally, there was a lack of fundamental demographic information and clinical presentation data. This constraint prohibited us from further investigating the causal association between gut microbiota and COPD at the species level. Secondly, the lowest taxonomic level in the exposure dataset was genus, which prevented us from investigating the relationship at the species level. Thirdly, the number of SNPs that could be studied further after being obtained based on a genome-wide statistical significance threshold of 5 × 10^−8^ was insufficient. Because of this, we only included the SNPs that achieved the required level of significance over the entire locus (1 × 10^−5^). These constraints limit the generalizability of the results and may compromise the study’s accuracy. Finally, although we conducted an extensive literature review and identified some confounding factors, there may still be potential unknown confounding factors that could have an impact on the results. Therefore, more care should be taken in interpreting the results.

## 5. Conclusion

In conclusion, by performing a two-sample MR analysis using publicly available GWAS summary-level data, we assessed the causal impact of gut microbiota on COPD and identified potentially pathogenic flora for COPD development. This study may be useful for screening gut microbial-based metabolites and markers for early detection of COPD as non-invasive diagnostic or therapeutic targets.

## Data availability statement

The original contributions presented in the study are included in the article/Supplementary material, further inquiries can be directed to the corresponding authors.

## Ethics statement

Written informed consent was obtained from the individual(s) for the publication of any potentially identifiable images or data included in this article.

## Author contributions

XL and CL conceived, designed the study, and revised the manuscript. YW downloaded and analyzed the data, wrote the original draft of the manuscript, and was responsible for the data visualization. XL contributed to interpretation of the results. All authors have read and approved the final manuscript.

## Conflict of interest

The authors declare that the research was conducted in the absence of any commercial or financial relationships that could be construed as a potential conflict of interest.

## Publisher’s note

All claims expressed in this article are solely those of the authors and do not necessarily represent those of their affiliated organizations, or those of the publisher, the editors and the reviewers. Any product that may be evaluated in this article, or claim that may be made by its manufacturer, is not guaranteed or endorsed by the publisher.

## Footnotes

^1^ https://mibiogen.gcc.rug.nl
