# Corrigendum: Gut microbiota and chronic obstructive pulmonary disease: a Mendelian randomization study

**DOI:** 10.3389/fmicb.2023.1335133

**Published:** 2023-11-29

**Authors:** Yi Wei, Xuechao Lu, Chao Liu

**Affiliations:** ^1^Department of Chinese Medicine, Shandong University of Traditional Chinese Medicine, Jinan, China; ^2^Department of Respiratory and Critical Care Medicine, Qingdao Traditional Chinese Medicine Hospital (Qingdao Hiser Hospital), Qingdao, China; ^3^Department of Medical Imaging, Qingdao Traditional Chinese Medicine Hospital (Qingdao Hiser Hospital), Qingdao, China

**Keywords:** gut microbiota, chronic obstructive pulmonary disease, mendelian randomization, causal association, genome-wide association study

In the published article, there was an error in Results, *3.1 Characteristics of SNPs*, Paragraph One. This sentence previously stated:

“A total of 2,571 SNPs were associated with COPD after excluding the effect of LD in specific bacterial groups.”

The corrected sentence appears below:

“A total of 2,561 SNPs were associated with COPD after excluding the effect of LD in specific bacterial groups.”

A correction has been made to Methods, *2.2 Ethics statement*, Paragraph One. Our original expression was ambiguous and has been changed for clarity. This sentence previously stated:

“All studies were conducted in accordance with the Declaration of Helsinki and were conducted with the approval of the appropriate institutional ethics committees, and therefore did not require additional ethical approval.”

The corrected sentence appears below:

“All original studies were conducted in accordance with the Declaration of Helsinki and were conducted with the approval of the relevant ethics committees (MiBioGen Consortium and FinnGen research consortium). This study only used publicly available summary-level data from published studies, therefore did not require additional ethical approval.”

In the published article, there was an error in Figure 2 as published. We carelessly omitted to display the result of family Victivallaceae in the forest plot. This result was not omitted in the article or in the Supplementary material, and the legend of Figure 2 is correct. Therefore, only Figure 2 needs to be updated. The corrected Figure 2 and its caption appear below.

**Figure 2:**
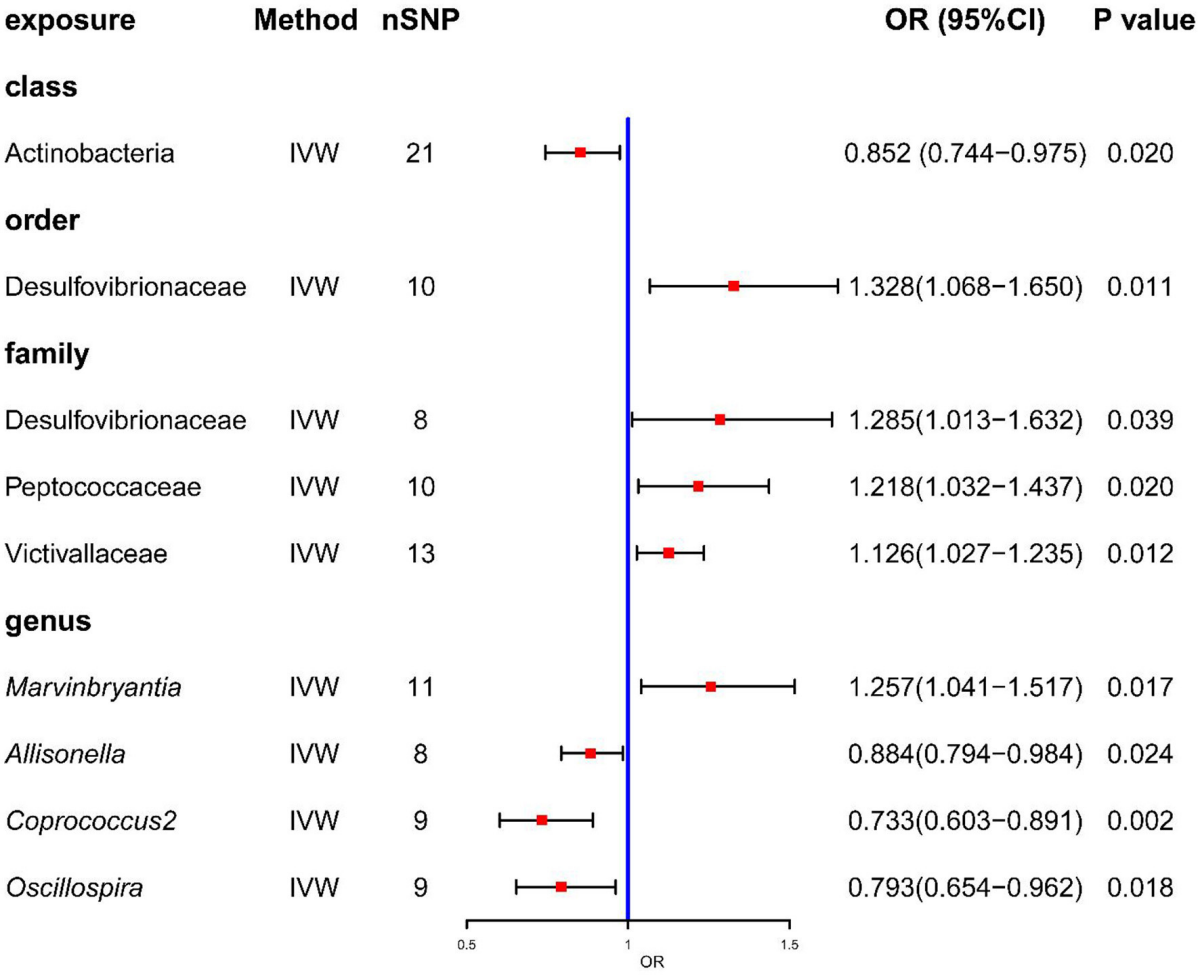
Forrest plot for summary causal effects of gut microbiota on COPD risk based on IVW method for the primary analysis. The forest plot demonstrates that class Actinobacteria, order Desulfovibrionales, family Desulfovibrionaceae, family Peptococcaceae, family Victivallaceae and genus *Marvinbryantia*, genus *Allisonella*, genus *Coprococcus2* and genus *Oscillospira* have causal effect on COPD risk.

The authors apologize for this error and state that this does not change the scientific conclusions of the article in any way. The original article has been updated.

