# Supplementary material for: Gut microbiota and chronic obstructive pulmonary disease: a Mendelian randomization study
Source: Front Microbiol. 2023 Jun 19;14:1196751. doi: 10.3389/fmicb.2023.1196751 (PMC10315658; doi:10.3389/fmicb.2023.1196751)
Supplement: Supplementary file 2 [file Data_Sheet_1.docx]

**Supplementary Figures**

**Supplemental Figure 1.** The scatter plots the 5 MR approaches for association between gut microbiota and COPD.

**Supplemental Figure 2.** The forest plots for the association between gut microbiota and COPD.

**Supplemental Figure 3.** The leave-one-out sensitivity analysis for the association between gut microbiota and COPD.

**Supplemental Figure 1. The scatter plots for association between gut microbiota and COPD.**


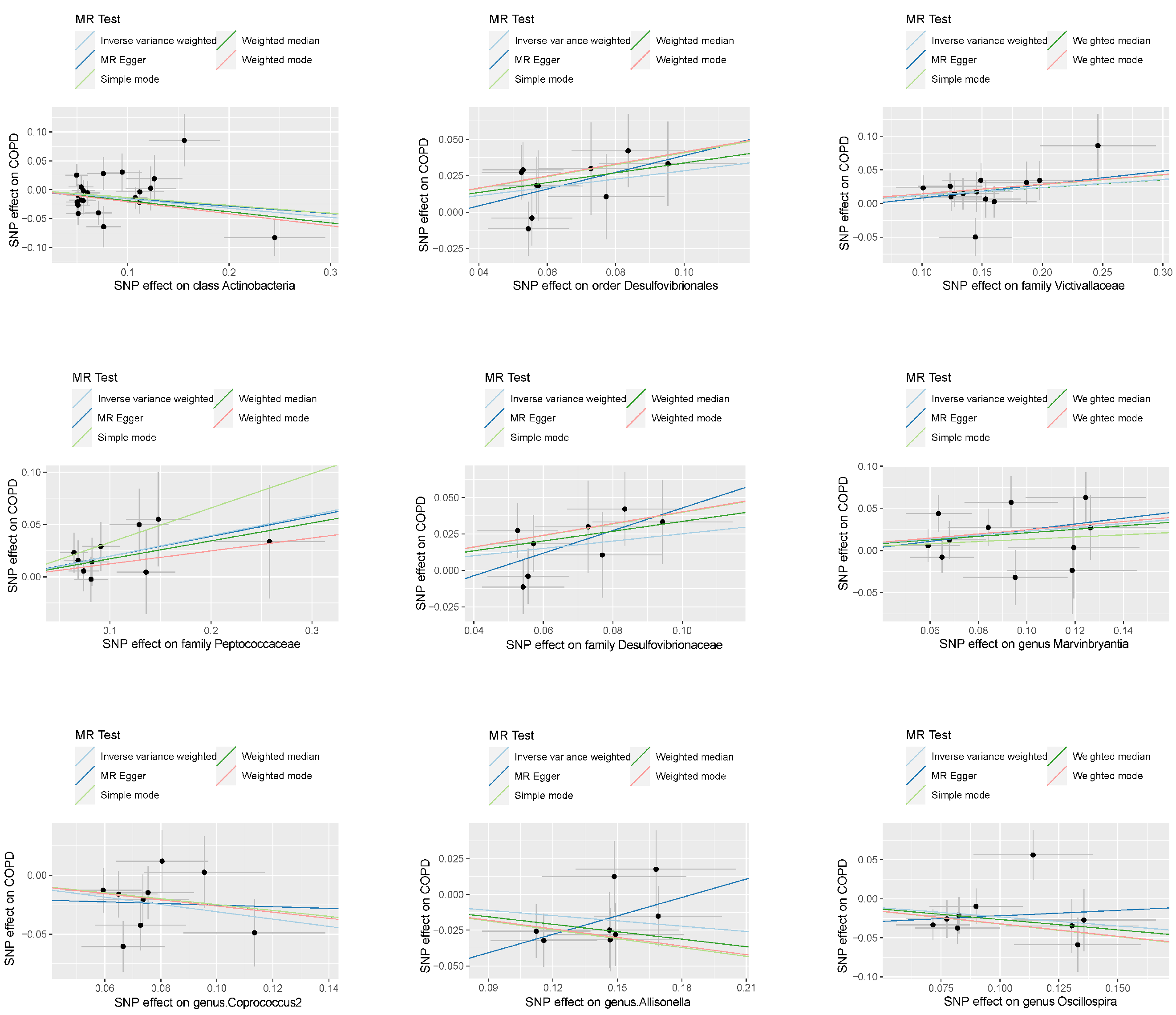


SNP effects were plotted into lines for the inverse-variance weighted test (light blue line), MR-Egger (blue line), weighted median (green line), Simple mode (light green line) and Weighted mode (red line). The slope of the line corresponded to the causal estimation.

**Supplemental Figure 2. Forest plots for causal effects of gut microbiota on COPD risk with individual SNPs.**


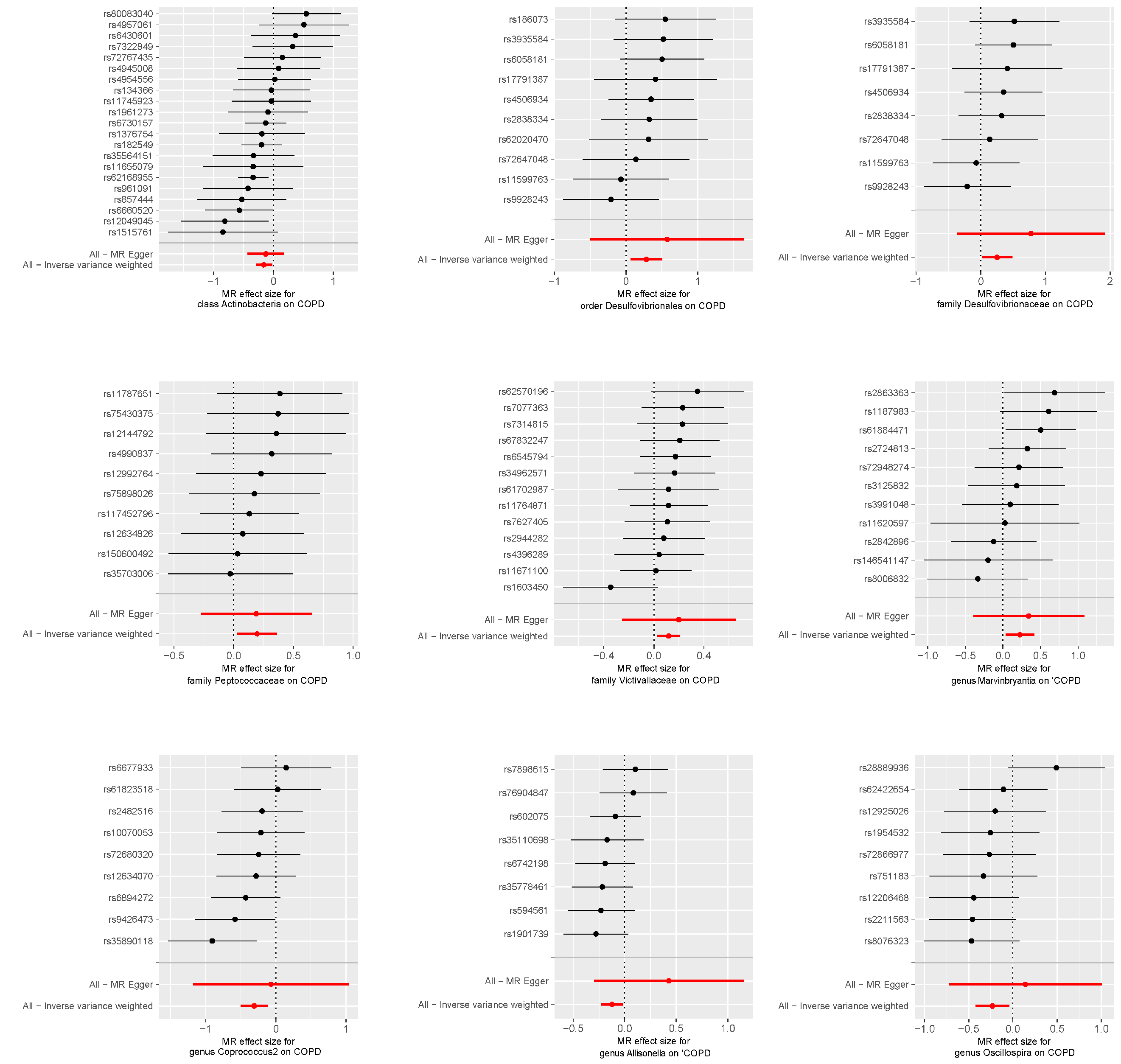


Black points denote the effect estimates of class Actinobacteria, order Desulfovibrionales, family Desulfovibrionaceae, family Peptococcaceae, family Victivallaceae and genus *Marvinbryantia,* genus *Allisonella*, genus *Coprococcus2* and genus *Oscillospira* on the risk of COPD using single SNPs, and the black lines signify the 95% CIs of the estimates. The red points symbolize overall effect estimates of the gut microbiota using the Egger and IVW method, and the red lines indicate their 95% CIs**.**

**Supplemental Figure 3. Plots for "leave-one-out" analysis for causal effect of gut microbiota on COPD risk.**


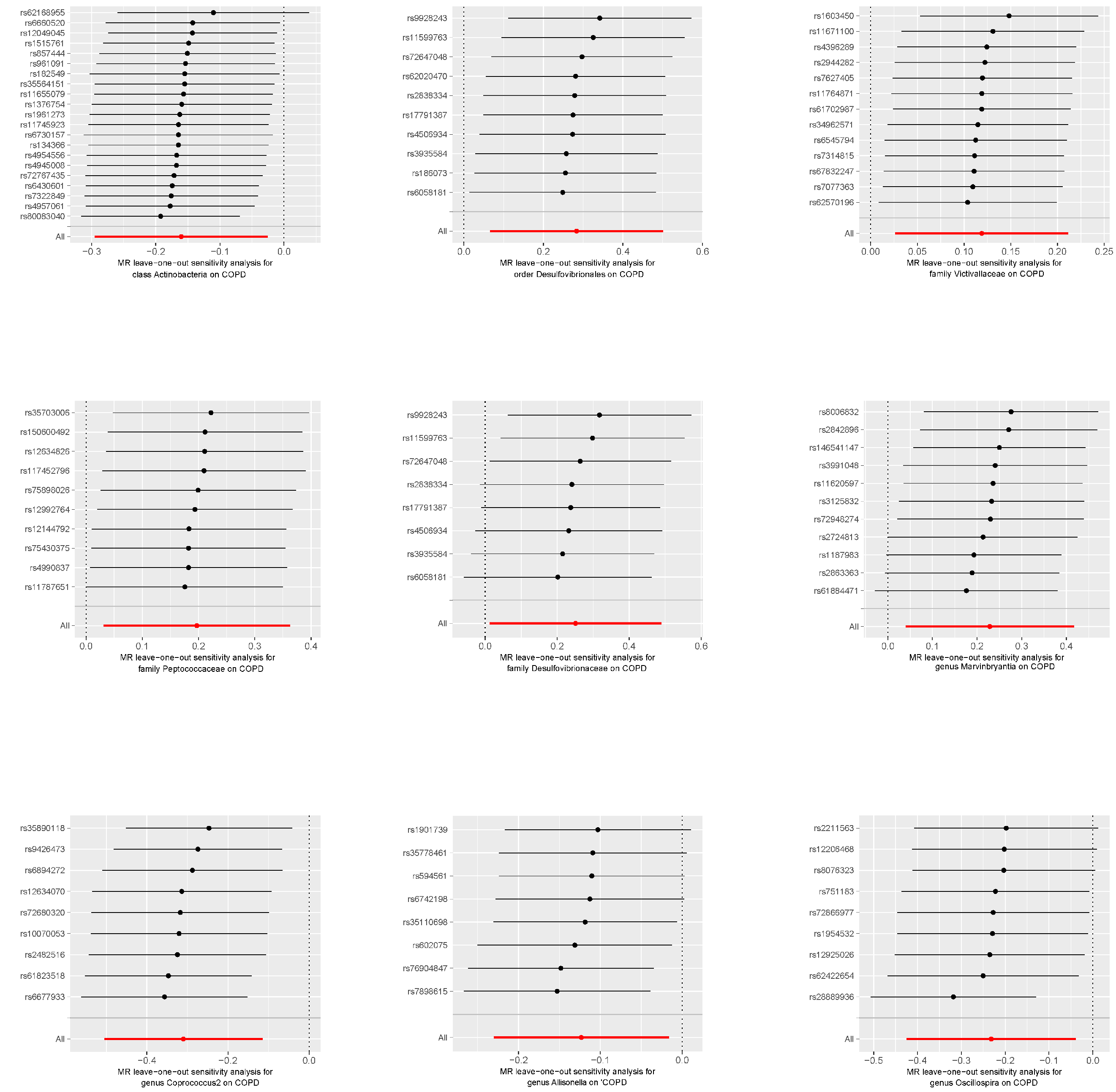


**Plots for "leave-one-out" analysis** **for causal effect of gut microbiota on COPD risk.** Leave-one-out plot helps determine whether the overall effect is altered by one or more specific genetic variants by sequentially re-evaluating causal estimates after discarding one SNP at a time. The four plots reveal no single SNP distorting overall MR estimates when each SNP is removed from principal MR analyses. The black points denote effect estimates of the gut microbiota after discarding a certain SNP, and black lines signify the corresponding 95% CIs of estimates. Red points symbolize overall causal effect estimate of the gut microbiota on COPD risk using a set of SNPs, and red lines indicate the corresponding 95% CIs.
